# Geometry Optimization Algorithms in Conjunction with the Machine Learning Potential ANI-2x Facilitate the Structure-Based Virtual Screening and Binding Mode Prediction

**DOI:** 10.3390/biom14060648

**Published:** 2024-05-31

**Authors:** Luxuan Wang, Xibing He, Beihong Ji, Fengyang Han, Taoyu Niu, Lianjin Cai, Jingchen Zhai, Dongxiao Hao, Junmei Wang

**Affiliations:** 1 Department of Pharmaceutical Sciences, Computational Chemical Genomics Screening Center, School of Pharmacy, University of Pittsburgh, Pittsburgh, PA 15261, USA; luw49@pitt.edu (L.W.); xibing.he@pitt.edu (X.H.); bej22@pitt.edu (B.J.); feh34@pitt.edu (F.H.); tan77@pitt.edu (T.N.); lic154@pitt.edu (L.C.); jiz183@pitt.edu (J.Z.); 2 School of Electronics and Information Engineering, Ankang University, Ankang 725000, China

**Keywords:** docking protocol, ANI-2x potential, virtual screening

## Abstract

Structure-based virtual screening utilizes molecular docking to explore and analyze ligand–macromolecule interactions, crucial for identifying and developing potential drug candidates. Although there is availability of several widely used docking programs, the accurate prediction of binding affinity and binding mode still presents challenges. In this study, we introduced a novel protocol that combines our in-house geometry optimization algorithm, the conjugate gradient with backtracking line search (CG-BS), which is capable of restraining and constraining rotatable torsional angles and other geometric parameters with a highly accurate machine learning potential, ANI-2x, renowned for its precise molecular energy predictions reassembling the wB97X/6-31G(d) model. By integrating this protocol with binding pose prediction using the Glide, we conducted additional structural optimization and potential energy prediction on 11 small molecule–macromolecule and 12 peptide–macromolecule systems. We observed that ANI-2x/CG-BS greatly improved the docking power, not only optimizing binding poses more effectively, particularly when the RMSD of the predicted binding pose by Glide exceeded around 5 Å, but also achieving a 26% higher success rate in identifying those native-like binding poses at the top rank compared to Glide docking. As for the scoring and ranking powers, ANI-2x/CG-BS demonstrated an enhanced performance in predicting and ranking hundreds or thousands of ligands over Glide docking. For example, Pearson’s and Spearman’s correlation coefficients remarkedly increased from 0.24 and 0.14 with Glide docking to 0.85 and 0.69, respectively, with the addition of ANI-2x/CG-BS for optimizing and ranking small molecules binding to the bacterial ribosomal aminoacyl-tRNA receptor. These results suggest that ANI-2x/CG-BS holds considerable potential for being integrated into virtual screening pipelines due to its enhanced docking performance.

## 1. Introduction

Molecular docking is a vital pipeline for computer-aided drug design (CADD), serving purposes from hit identification to lead optimization. Docking relies on scoring functions to quantitatively assess the chemical and steric compatibility between ligands and macromolecules, thus attempting to predict the experimental binding mode of the ligand at the specified binding site and corresponding binding affinity [1,2]. The classic scoring functions can be broadly grouped into three categories: empirical methods (e.g., Glide Score and ChemScore), knowledge-based methods (such as IT-Score and Drugscore), and force field-based methods (for example DOCK). These classifications are grounded on the essential energetic components of protein–ligand binding, the statistical potential derived from known protein–ligand complexes, and molecular mechanical calculations that inform binding affinity predictions [3,4]. The evaluation of scoring functions in molecular docking is critical, focusing on various key performance metrics, as the success of molecular docking is intricately linked to the quality of these functions, driving continuous efforts for innovation and improvement. These metrics include (1) docking power, which evaluates the scoring function’s capability to accurately predict and detect the native binding mode of the ligand among the decoys generated by the sampling algorithm; (2) ranking power, which refers to the ability to correctly rank known entities based on their binding scores; (3) screening power, which describes the proficiency of a scoring function in identifying genuine binders from libraries of random molecules; and (4) scoring power, which indicates the degree to which the binding affinity predictions made by a scoring function linearly correlate with the experimental results [5,6]. As scoring functions continue to evolve, extensive comparative studies reveal that their performance in docking power is generally acceptable. However, there is still room for improvement in screening, ranking, and scoring powers [6,7,8]. At the same time, molecular docking, unlike the rigorous prediction of binding affinity, does not require extensive sampling of complex conformations or detailed treatment of the aqueous solution environment. It primarily relies on the structure of a single ligand–macromolecule complex, which enables high efficiency when screening large molecular libraries for potential drug candidates, a common practice in structure-based virtual screening (SBVS) [9]. However, this approach can lead to inaccurate binding affinity predictions due to limited sampling space and an increased likelihood of identifying false-positive hits as the size of the molecular library expands [10]. Therefore, in light of the above considerations and with the aim of enhancing the docking, scoring, and ranking powers in molecular docking, we proposed an innovative docking pipeline, which combines our unique geometry optimization algorithm, the conjugate gradient with backtracking line search (CG-BS), alongside the highly precise machine learning potential, ANI-2x, which emulates the accuracy of the model chemistry at the wB97X/6-31G(d) level. We integrated this protocol in the binding pose prediction process to improve the overall efficacy and accuracy of molecular docking by a mainstream docking program: Glide implemented in the Schrodinger 2017 software package (Schrodinger Inc. New York, NY, USA).

The initial version of the Accurate NeurAl networK engINe for Molecular Energies (ANI) is the first truly transferable neural network-based atomistic potential for organic molecules [11]. Drawing from a dataset enriched with varied molecular conformations of molecules containing carbon (C), hydrogen (H), oxygen (O), and nitrogen (N), ANI potential is capable of simulating the potential energy surface (PES) of an arbitrary molecular system consisting of the aforementioned four elements. It achieved a result with a fidelity comparable to quantum mechanical methods but at a substantially lower computational overhead. Building on this foundational work, ANI-2x further integrated sulfur (S), fluorine (F), and chlorine (Cl) elements. This expansion allows ANI-2x to address a more extensive range of organic chemical phenomena, rendering it an optimal choice for comprehensive molecular dynamics studies and small-molecule drug design [11,12,13]. Given the accuracy and efficiency of ANI-2x in predicting energy and force, it becomes an appealing choice for geometry minimization tasks. However, the PESs generated by machine learning models like ANI-2x tend to be less smooth than those ab initio potentials. Even if we integrated Gaussian16 with ANI potential for geometry optimization, it led to issues like non-convergence or CPU inefficiencies. To counteract this, we have innovated four optimization algorithms tailored for the ANI potential [14]. By harnessing the energy and forces produced through the ANI, these algorithms successfully bolster ANI’s capability in geometry minimization and yield more consistent PESs compared with density functional theory results. Among the quartet of algorithms, the conjugate gradient backtracking line search (CG-BS) especially stands out, which adeptly incorporates previous movement directions and ensures efficient iteration pacing by adhering to Wolfe conditions, demonstrating effective and robust results combined with both ANI-1x and ANI-2x potentials [14,15].

In this study, we integrated our newly proposed ANI-2x/CG-BS protocol with the module Glide [16,17] implemented in the Schrodinger Suite (Maestro 11.2), where ANI-2x/CG-BS employs the binding pose generated by the Glide as the initial structure for further geometric optimization and binding energy prediction. Through testing on a total of 11 small molecule–macromolecule systems and 12 peptide–macromolecule systems, our novel protocol showed enhancements in the docking, scoring, and ranking stages. Consequently, considering the observed improvements, it is conceivable to integrate our approach into virtual screening workflows to enhance high-throughput virtual screening (HTVS) and facilitate binding mode predictions. This integration holds promise for drug lead identification and advancing drug discovery processes.

## 2. Methods

### 2.1. Workflow Overlook

**Figure 1:**
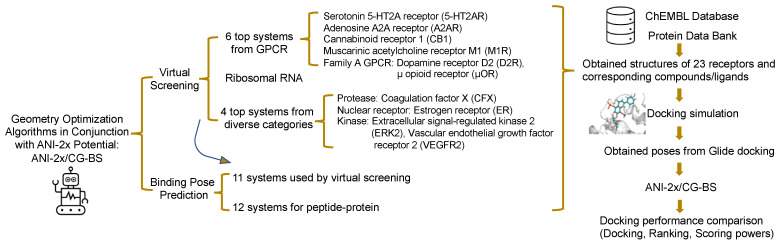
The comprehensive workflow.

In this work, we utilized a total of 11 small molecule–macromolecule systems and 12 peptide–macromolecule systems to assess the feasibility of our new pipeline and its potential to enhance the performance of docking. For those 11 diverse small molecule–macromolecule systems, we acquired the X-ray structures of receptors from the Protein Data Bank (PDB) [18] and obtained experimental data on small molecules along with their 3D structure from the ChEMBL database [19]. This compilation was designated as Dataset 1 and used for virtual screening to assess the scoring and ranking powers. For Dataset 2, we gathered the corresponding ligands with X-ray structures for the aforementioned 11 systems’ receptors and additionally incorporated 12 peptide–protein systems, with the X-ray structures for both peptides and proteins sourced from the PDB. This dataset was designed to evaluate the docking power. We subsequently harnessed Glide to undertake docking on those systems, with the ensuing docking poses setting the preliminary conformations for ANI-2x/CG-BS. These structures then underwent optimization and PES evaluations through ANI-2x/CG-BS. In the final analysis, the predictions from both Glide and ANI-2x/CG-BS were juxtaposed with empirical findings. The comprehensive workflow is represented in Figure 1. In-depth methods will be expounded in subsequent sections.

### 2.2. Datasets Preparation

We used 11 small molecule–macromolecule systems collected from our previous studies [20] in the ChEMBL database as our Dataset 1. All the receptor categories are selected based on the ranking of member counts within diverse target categories and the selection of specific targets was influenced by the number of compounds documented in binding assays, resulting in 11 diverse receptors from varied categories such as GPCR, kinase, protease, nuclear receptors, and RNA. The X-ray structures of all receptors were obtained from PDB. Detailed information on these receptors can be found in Table 1. Regarding the 3D structure of the small molecules, they were collected based on their Ki values recorded from binding assays, except for the ribosomal RNA (rRNA) receptor, for which we relied on Kd values due to the scarcity of Ki records. To ensure a representative distribution of Ki values across receptors, we selected up to 300 compounds from each of the four distinct Ki tiers: Ki < 10 nM, 10 nM ≤ Ki < 1 μM, 1 μM ≤ Ki < 100 μM, and Ki ≥ 100 μM. We excluded compounds that have very weak binding affinity (a binding energy above −4 kcal/mol) to enhance our evaluation’s reliability. Given that the ANI-2x potential is tailored for molecules with C, H, O, N, S, F, and Cl atoms, we further filtered out compounds with other atomic constituents. Comprehensive details of the selected small molecules are provided in Supporting Information Table S1.

For Dataset 2, we collected the corresponding natural binding ligands with X-ray structures for the 11 diverse receptors instead of gathering compounds with binding assay records from the ChEMBL database. Specifically, in the context of the CB1 system, we opted for a different PDB ID (6KQI) for the CB1 receptor due to the presence of a bromine (Br) atom in the ligand associated with the initially selected CB1 receptor, which the ANI-2x potential cannot effectively accommodate. Additionally, we also introduced 12 more flexible peptide–protein systems of varying lengths randomly selected from the LEADS-PEP Benchmark Dataset [21,22]. It is pointed out that we did not select peptide–protein systems from different drug target families, given the fact that there are not many approved peptide drugs. The X-ray crystal structures of all the ligands and receptors used in our study for these 23 systems were sourced from the PDB and detailed information can be found in Table 1.

### 2.3. Structure Preparation and Docking

We followed standard protocols to prepare structures, generate grid files, and conduct flexible docking, as detailed below. For all systems under study, the water molecules, cofactors, and ions were preliminarily removed. Ligands were prepared with AMBER’s LEaP [23] and Maestro’s Ligprep programs [24], adding necessary hydrogen atoms, completing atomic structures, and determining protonation and tautomeric states according to the experimental pH. Receptor preparation was carried out via Maestro’s Protein Preparation Wizard module [25], where we added the missing atoms and conducted energy minimization of the hydrogens. The docking grid box was centered on the coordinates of the naturally bound ligand, with the size of the box chosen to accommodate the ligand of a similar size to the natural binder. Flexible docking was executed with a standard precision scoring function, where we manually rewarded intramolecular hydrogen bonds and enhanced the planarity of conjugated pi groups while maintaining default settings for all other parameters. Finally, the top 10 docking poses for each ligand were recorded.

### 2.4. ANI-2x/CG-BS Calculation

The new docking pipeline ANI-2x/CG-BS utilizes the precise molecular energy and force predictions provided by the ANI-2x machine learning potential, which emulates the wB97X/6-31G(d) level of theory, to assist the CG-BS algorithm in iteratively updating the molecular coordinates until convergence is achieved. For ANI-2x/CG-BS application, initial preprocessing of the receptor is crucial. We focused on residues within an 8–15 Å radius from the center of the naturally bound ligand to simplify the receptor while maintaining its interaction potential. Building on this, we defined a boundary at 2.5 Å from any ligand atoms: the exterior region becomes the “frozen part” with a temperature coefficient set to −1 kcal/mol/Å^2^, indicating it remains static during the optimization, and the interior region is the “active part” with a temperature coefficient of 5 kcal/mol/Å^2^, allowing for restrained movement. We combined the processed receptor with the ligand pose generated from Glide docking to form the complex. The entire complex is then subjected to ANI-2x/CG-BS optimization and PES evaluation. An ANI-2x/CG-BS optimization is converged when it satisfies the same criteria used by Gaussian 16, i.e., maximum force and RMS force are 0.00045 and 0.0003 atomic units, and maximum displacement and RMS displacement are 0.0018 and 0.0012 atomic units. Upon obtaining the final predicted individual energies of the ligand, receptor, and complex, we applied the following formula to calculate the binding energy:

ΔEbind=Ecom−Erec−Elig

### 2.5. Evaluation Metrics

#### 2.5.1. Root-Mean-Square Deviation (RMSD)

The RMSD functions as a key indicator for determining the average variation in position between matching atoms, often with a focus on the backbone atoms, across two molecular formations. It plays a crucial role in quantifying how similar two conformational states of a molecule are. In the context of docking simulations, RMSD is commonly employed to pinpoint the conformation that most accurately mirrors the native structure amid a broad array of docked configurations [26,27]. In this study, considering assessing both the position and orientation of a ligand within its active site, the fitting alignment is anchored to the receptor. Ligands only undergo coordinate adjustments based on the receptor alignment matrix, while avoiding direct fitting to their crystal structures: by doing so in addition to conformational changes, the translational and rotational movement of the ligand in the binding pocket were also counted. This metric was used for docking power evaluation.

#### 2.5.2. Pearson’s Correlation Coefficient (R)

Pearson’s correlation coefficient is a statistical tool used to evaluate the degree of a linear connection between two variables. The coefficient R-value fluctuates from −1 to 1. A positive or negative sign of R signifies the nature of the relationship: positive or negative, respectively. When the absolute value of the R approaches 1, it implies a strong correlation, suggesting a high degree of similarity between the variables. Conversely, an absolute value close to 0 indicates a lack of correlation, meaning there is little to no linear relationship between the variables. This metric was used for scoring power evaluation.

#### 2.5.3. Spearman’s Rank Correlation Coefficient (ρ)

Spearman’s rank correlation coefficient is a statistical method focusing on the rank order of data across two variables. It ranks the data within each variable before computing Pearson’s correlation coefficient for these ranks. This approach is less influenced by outliers and is especially effective in determining the strength and direction of monotonic relationships. In such relationships, the variables consistently move in the same direction, either increasing or decreasing, but not necessarily at a steady rate. The values of this coefficient span from −1 to 1. A ρ value near 1 in absolute terms suggests a complete rank association. On the other hand, an absolute value nearing 0 implies a lack of any rank association [28]. This metric was used for ranking power evaluation.

## 3. Results

### 3.1. Docking Power Evaluation

Although many comparative studies have demonstrated that docking programs often exhibit a better docking power compared to their screening and ranking powers, it remains crucial to emphasize the significance of precise prediction of the native ligand binding mode in docking simulation. It is understandable that the actual docking power is lower when it is evaluated using ligands without forming crystallized complex structures. If a docking program is unable to produce a pose that closely aligns with the natural pose and to accurately prioritize such native-like binding poses at the forefront, the predicted binding affinity will become less reliable, and the selection of the most accurate binding pose based solely on its score will be less precise. As a result, the subsequent ranking and screening processes, which rely on these affinity predictions, will be inherently flawed. Hence, in our study, we applied ANI-2x/CG-BS to Dataset 2 with the hope of enhancing docking power beyond the current capabilities of Glide. Dataset 2 includes receptors and their corresponding naturally binding ligands across 11 small molecule–macromolecule systems and 12 peptide–protein systems. Peptides differ from small molecules in that they are considerably larger, and their numerous rotatable bonds confer a higher degree of flexibility. Furthermore, the sites at which they bind are often superficial and exposed to solvents [21]. Thus, we included these 12 peptide–protein systems to evaluate if our newly developed protocol is capable of dealing with systems that are typically challenging for the Glide docking program. We obtained the X-ray structures for all the receptors and ligands from the PDB to directly perform docking and generated ten binding poses, which served as the starting point for further optimization and energy prediction by ANI-2x/CG-BS. Following the generation of poses through docking and their subsequent refinement with our new docking pipeline, we ranked them according to their docking scores and predicted energies, so that the Top 1 pose has the best docking score/predicted energy and so on. We then calculated the RMSD of each pose compared to the ligand’s crystal structure. The lowest RMSDs were evaluated within four ranking-based categories: Top 1, Top 3, Top 5, and Top 10. If, across most systems, the lowest RMSD among the top 10 poses is frequently found within the Top 1 or Top 3 categories post-ANI-2x/CG-BS optimization, and if this RMSD is smaller than that of poses obtained through Glide docking, it would indicate an improvement performance of the ANI-2x/CG-BS methods in predicting and identifying native-like poses from decoys. Table 2 summarizes the lowest RMSD values belonging to the four ranking-based categories of docking poses obtained from Glide docking and ANI-2x/CG-BS optimization conducted after Glide docking. 

**Figure 2:**
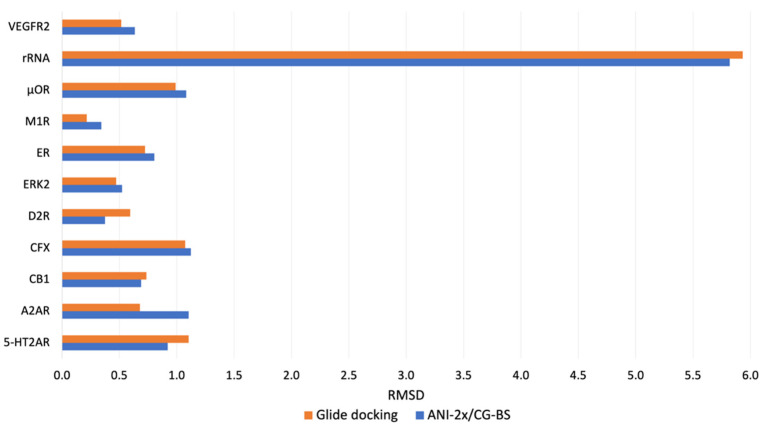
Comparison of the lowest RMSD results among the top 10 docking poses obtained from Glide docking and ANI-2x/CG-BS optimization methods for the 11 small molecule–macromolecule systems.

**Figure 3:**
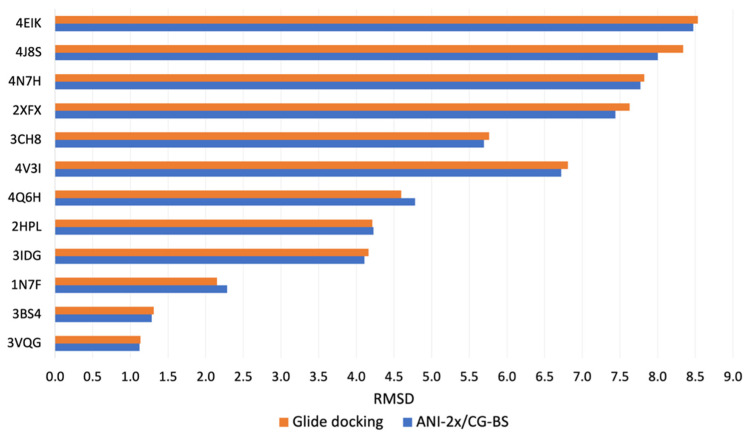
Comparison of the lowest RMSD results among the top 10 docking poses obtained from Glide docking and ANI-2x/CG-BS optimization methods for the 12 peptide–protein systems.

Figure 2 and Figure 3 specifically compare the RMSD between the most native-like structures of ligands obtained through two different methods, namely Glide docking and the newly proposed docking pipeline, for the 11 small molecule–macromolecule systems and 12 peptide–protein systems, respectively, against their corresponding crystal structures. In Figure 2, which pertains to the 11 small molecule–macromolecule systems, the smallest RMSD values from Glide docking almost consistently remain under 1.5 Å except for the rRNA system. After optimization, a slight reduction in RMSD is observed in the 5-HT2AR, CB1, D2R, and rRNA systems. In the case of the remaining systems, even though there are minor increases in RMSD post-optimization, the smallest RMSD values for these optimized poses still typically fall below the 1.5 Å threshold. In the case of the 12 peptide–protein systems, as depicted in Figure 3, we observed a distinct advantage of ANI-2x/CG-BS optimization when the overall accuracy of Glide docking decreased. Specifically, when the pose with the lowest RMSD generated by Glide docking exceeds around 5 Å, ANI-2x/CG-BS optimization tends to reduce the RMSD. For instance, in the system with PDB ID 4J8S, the RMSD of the pose closest to the true structure initially obtained from docking was 8.34 Å, which, after optimization, decreased to 8.00 Å, showing a change of 0.34 Å. Furthermore, in systems where Glide docking already predicts poses accurately, optimization can still lead to a reduction in RMSD or maintain comparable accuracy, like those with PDB IDs 3VQG and 3BS4.

In assessing the proficiency of different docking methods to prioritize native-like binding poses, our analysis focused on the frequency of these poses being ranked within four predefined categories (Table 2). With Glide docking, the native-like pose was found to be ranked at the top position in 11 of the systems studied, with half being accurately identified based on the best docking score as Top 1, while the other half were identified at the second or third ranks. However, in the remaining 12 systems, these native-like poses were predominantly identified in lower-ranking categories. The ANI-2x/CG-BS method, however, showed a stronger performance, identifying native-like poses within the top three poses in 17 systems. Only in six systems were the native-like poses primarily identified in lower-ranking categories, highlighting the superior capability of ANI-2x/CG-BS in pinpointing the pose that closely resembles the actual native binding configuration from computational decoys.

A strong docking power refers to the ability to not only accurately predict binding poses but also to discern native-like binding poses from computational decoys. Based on our results, when the binding poses generated by Glide docking closely match the native ligand conformation, the subsequent optimization by ANI-2x/CG-BS tends to offer limited improvement, often maintaining similar RMSD levels. However, in instances where the binding poses from Glide docking significantly deviate from the native ligand structure, post-optimization with ANI-2x/CG-BS effectively refines these poses, bringing them closer to the true binding pose, with RMSD reductions around 0.2 Å. In terms of identifying native-like binding poses, the ANI-2x/CG-BS method generally outperforms Glide docking. The analysis reveals that ANI-2x/CG-BS can identify native-like poses within the top three poses in 74% of the analyzed systems, whereas Glide docking achieves this in only 48%. It is pointed out that in real practice of HTVS, ANI-2x/CG-BS has a more significant advantage over Glide as most small molecular ligands do not have crystalized complex structures at all. Therefore, integrating ANI-2x/CG-BS as a follow-up optimization step to Glide docking can enhance the overall docking power, improving the accuracy and reliability of the docking predictions.

### 3.2. Ranking and Scoring Power Evaluations

Dataset 1 was employed for assessing and contrasting the ranking and scoring powers of the Glide method against our innovative ANI-2x/CG-BS approach. This dataset includes a variety of 11 distinct small molecule–macromolecule systems, encompassing receptor classes such as GPCR, kinase, RNA, protease, and nuclear receptors, together with their respective, selected compounds sourced from the ChEMBL database. Standard precision Glide docking was executed to determine the best docking poses, which subsequently served as the reference for additional optimization via our newly formulated docking pipeline. The comparative scoring and ranking powers of these two methods were quantified using Pearson’s correlation coefficient (R) and Spearman’s correlation coefficient (ρ) as the evaluative metrics, respectively. The outcomes of these comparative assessments are detailed in Table 3 and Table 4.

Table 3 presents the Pearson’s correlation coefficients for those 11 small molecule–macromolecule systems. It provides a measure of the correlation between the Glide docking scores and binding energies derived for the ANI-2x/CG-BS optimized approach, as compared to experimental binding affinity. Notably, among the 11 systems studied, four systems (CB1, D2R, M1R, and rRNA) showed an improvement in their R-values after optimization with ANI-2x/CG-BS. The most significant improvement was observed in the rRNA system, where the scoring power, represented by R, increased markedly from 0.24 to 0.85. For the 5-HT2AR, ER, μOR, and VEGFR2 systems, the scoring power was found to be comparable between the two methods, with no significant changes observed. However, for the A2AR, CFX, and ERK2 systems, the scoring power underperformed compared to Glide docking. When averaging the R-values across all 11 systems, the overall performance of our newly proposed ANI-2x/CG-BS docking pipeline showed an improvement over Glide docking alone, with the average R-value increasing from 0.29 to 0.32. Table 4 summarizes the ranking power of each system as characterized by Spearman’s correlation coefficient (ρ). The trend in ranking power largely mirrors that of the scoring power observed previously. Following the optimization with the ANI-2x/CG-BS method, five of the systems (CB1, D2R, M1R, μOR, and rRNA) exhibited an improvement in their ρ values, indicating enhanced ranking power. Conversely, for the A2AR, CFX, and ERK2 systems, the ranking power notably declined compared to Glide docking after implementing this new pipeline. When averaging the results across all 11 systems, it can be generally stated that the newly introduced docking pipeline did enhance the ranking power, albeit with a modest increase in the ρ value of 0.01.

In summary, integrating the ANI-2x/CG-BS pipeline with Glide docking demonstrates the promising potential to enhance ranking and scoring powers compared to using Glide docking alone for HTVS. Among the 11 molecular systems analyzed, while maintaining comparable results in 20% of cases, this integrated approach showed improvements in ranking and scoring powers in nearly half of the systems. This indicates a slight advancement in the method’s ability to accurately predict and rank molecular interactions despite the performance being decreased for three systems. The most noteworthy improvement was observed in the rRNA system, where both ranking and scoring powers significantly increased post-optimization. This highlights the potential of the ANI-2x/CG-BS pipeline in enhancing the predictive accuracy and efficiency of molecular docking in complex systems.

## 4. Discussion

This study introduces ANI-2x/CG-BS, a novel docking pipeline applied to post-Glide docking for binding pose refinement and binding affinity re-ranking. The CG-BS optimization algorithm utilizes the precise potential energies and forces provided by the ANI-2x machine learning potential to iteratively optimize the poses generated by Glide until convergence is achieved. By applying this additional optimization protocol to 11 diverse small molecule–macromolecule systems and 12 flexible peptide–protein systems, we observed an improvement in the docking power over Glide docking across most scenarios. When Glide docking accurately predicts binding poses, the introduction of ANI-2x/CG-BS results in minimal changes. However, in systems with greater flexibility that are challenging for standard Glide docking, our proposed method optimizes binding poses more reasonably. Furthermore, ANI-2x/CG-BS demonstrates remarkable capabilities in accurately identifying “native-like” binding poses, surpassing Glide docking by 26% in successful recognitions. Regarding scoring and ranking powers, the introduction of this method enhances these aspects for nearly 50% of the systems, particularly showing substantial improvements in the rRNA system.

After ANI-2x/CG-BS optimization and rescoring, 17 out of 23 docking systems have their best RMSD poses among the top three poses, while there are only 11 systems for Glide docking. In a prospective study, one may consider experimental evidence, especially mutagenesis data, to identify the best poses. Alternatively, one may also apply more advanced free energy-based methods, such as MM-PBSA [29,30], to determine the best poses. As to how to choose compounds to test in a virtual docking screening study, we recommend choosing ones with the best binding energy. Nevertheless, the ANI-2x/CG-BS approach still fails to achieve a substantial enhancement in the overall docking performance across all tested systems. This limitation stems from the foundation of our proposed approach, which builds upon Glide docking and ANI-2x potential. Glide docking, with its primary focus on optimizing individual ligands, inadvertently neglects the influence of substantial conformational changes in the receptor during ligand binding. Furthermore, the grid-based approach of Glide restricts the exploration of conformational space during docking, thereby failing to fully uncover the spectrum of possible ligand conformations. Consequently, these factors contribute to modest improvements in binding mode prediction with our proposed optimization. In the case of the ANI-2x potential, it is currently solely grounded in the gas phase environment, omitting the incorporation of solvation effects in the prediction of binding energy. Moreover, the current potential was trained using the relatively small and simple molecules and the net charge information was not being explicitly considered. Thus, it is understandable that ANI-2x energy may be less accurate for druglike molecules, especially the charged ones. Indeed, we found that the mean errors increased from 1.03 kcal/mol for the neutral PDB ligands to 1.56 kcal/mol for the charged ones [15]. To overcome these limitations, the next generation of machine learning potentials, which broaden the chemical space to cover more elements (such as Br and I), incorporate the solvent effects in ab initio potential energy calculation, and explicitly consider the charge states of the input molecule, is expected to significantly enhance the accuracy of binding energy predictions and lead to significant advancements in our innovative SBVS pipeline across diverse systems.

Although there is a great potential that the ANI-2x successors could significantly improve the docking performance, we must not rule out that other docking protocols outperform our docking protocol using ANI-2x. We have demonstrated that machine learning-trained ligand-residue interaction profile scoring functions (IPSF) achieve a significantly better performance in docking screening [31]. However, the IPSF-based approach requires existing ligand binding affinity data to train the model. Other successful docking protocols include the ones applying Dock3.7 [32] and Autodock [33].

## 5. Conclusions

In our study, we introduced a novel docking pipeline by integrating our in-house geometry optimization algorithm CG-BS with the highly accurate machine learning potential, ANI-2x, known for its precision and efficiency in molecular energy and force predictions. The application of Glide docking for predicting binding poses in 11 diverse small molecule–macromolecule systems and 12 flexible peptide–protein systems, followed by subsequent optimization using ANI-2x/CG-BS with these predictions as initial structures, has confirmed that incorporating ANI-2x/CG-BS as a subsequent step has resulted in enhancements in docking, scoring, and ranking powers. Considering that CG-BS can adopt most if not all the current and future machine learning potentials, our new docking pipeline holds great promise for globally elevating overall docking performance, especially when more accurate and advanced machine learning potentials come into being.

## Figures and Tables

**Table 1:** Detailed information on X-ray structures of receptors and ligands in 23 systems.

	Receptor Name/Peptide Residue Number	PDB ID	Resolution
11 small molecule–macromolecule systems	5-HT2AR	6A93	3.00Å
	A2AR	3EML	2.60 Å
	CB1	5XRA/6KQI	2.80 Å/3.25 Å
	CFX	1NFU	2.05 Å
	D2R	6CM4	2.87 Å
	ERK2	6SLG	1.33 Å
	ER	3OS8	2.03 Å
	M1R	5CXV	2.70 Å
	μOR	5C1M	2.10 Å
	rRNA	2F4S	2.80 Å
	VEGFR2	2OH4	2.05 Å
12 peptide–protein systems	4	3VQG	1.35 Å
	3	3BS4	1.60 Å
	8	1N7F	1.80 Å
	6	3IDG	1.86 Å
	5	2HPL	1.80 Å
	6	4Q6H	1.90 Å
	5	4V3I	1.50 Å
	8	3CH8	1.90 Å
	11	2XFX	1.90 Å
	9	4N7H	1.70 Å
	12	4J8S	1.55 Å
	11	4EIK	1.60 Å

**Table 2:** Docking power evaluation with Glide docking and ANI-2x/CG-BS optimization methods: lowest RMSD values across four ranking-based categories of docking poses.

	Receptor Name /PDB ID	RMSD (Å)							
		ANI-2x/CG-BS				Glide Docking			
		Top 1	Top 3	Top 5	Top 10	Top 1	Top 3	Top 5	Top 10
11 small molecule–macromolecule systems	5-HT2AR	2.96	0.92	0.92	0.92	2.98	2.25	1.56	1.10
	A2AR	1.43	1.10	1.10	1.10	1.85	1.85	0.68	0.68
	CB1	0.88	0.69	0.69	0.69	0.82	0.74	0.74	0.74
	CFX	8.69	8.48	1.62	1.12	8.55	8.27	1.47	1.07
	D2R	5.34	0.38	0.38	0.38	1.98	0.59	0.59	0.59
	ERK2	0.88	0.52	0.52	0.52	0.47	0.47	0.47	0.47
	ER	1.46	1.15	1.13	0.81	1.12	1.12	1.12	0.72
	M1R	0.89	0.34	0.34	0.34	1.15	0.22	0.22	0.22
	μOR	1.20	1.08	1.08	1.08	1.08	1.07	0.99	0.99
	rRNA	5.92	5.83	5.82	5.82	6.11	5.96	5.96	5.93
	VEGFR2	0.96	0.63	0.63	0.63	0.52	0.52	0.52	0.52
12 peptide–protein systems	3VQG	1.21	1.12	1.12	1.12	1.14	1.13	1.13	1.13
	3BS4	1.33	1.28	1.28	1.28	1.31	1.31	1.31	1.31
	1N7F	2.29	2.29	2.29	2.29	2.15	2.15	2.15	2.15
	3IDG	6.82	6.82	4.46	4.11	6.97	4.16	4.16	4.16
	2HPL	4.30	4.23	4.23	4.23	4.27	4.21	4.21	4.21
	4Q6H	5.93	4.78	4.78	4.78	6.05	4.63	4.60	4.60
	4V3I	6.72	6.72	6.72	6.72	8.08	8.08	8.08	6.81
	3CH8	12.99	11.97	7.17	5.69	7.07	7.07	7.07	5.76
	2XFX	14.52	7.44	7.44	7.44	10.38	10.38	7.63	7.63
	4N7H	11.02	7.77	7.77	7.77	9.99	9.64	7.82	7.82
	4J8S	8.83	8.83	8.29	8.00	8.71	8.71	8.71	8.34
	4EIK	12.50	8.47	8.47	8.47	8.53	8.53	8.53	8.53
The number of systems with minimal RMSD occurrences in each category		2	15	1	5	5	6	5	7
Top rank identification success rate (%)		74%				48%			

**Table 3:** Scoring power evaluation with Glide docking and ANI-2x/CG-BS optimization methods.

	5-HT2AR	A2AR	CB1	CFX	D2R	ERK2	ER	M1R	μOR	rRNA	VEGFR2	Average
Docking	0.23	0.29	0.19	0.10	0.11	0.60	0.60	0.21	0.16	0.24	0.48	0.29
ANI-2x/CG-BS	0.20	0.02	0.23	0.04	0.12	0.54	0.60	0.31	0.16	0.85	0.45	0.32

**Table 4:** Ranking power evaluation with Glide docking and ANI-2x/CG-BS optimization methods.

	5-HT2AR	A2AR	CB1	CFX	D2R	ERK2	ER	M1R	μOR	rRNA	VEGFR2	Average
Docking	0.21	0.30	0.21	0.29	0.10	0.57	0.65	0.30	0.14	0.14	0.47	0.30
ANI-2x/CG-BS	0.19	0.07	0.25	0.06	0.14	0.36	0.61	0.47	0.16	0.69	0.46	0.31

## Data Availability

All the data were collected using databases (protein data bank and ChEMBL database) and software (Glide 2017 and Amber 22) in the public domain. ANI-2x/CG-BS code can be downloaded freely from https://github.com/junmwang/pyani_mmff.

## Supplementary Materials

The following supporting information can be downloaded at: https://www.mdpi.com/article/10.3390/biom14060648/s1, Table S1: the detailed docking score, inhibition constant, and experimental energies of selected compounds for all 11 diverse receptors.
